# Unlocking plant regeneration capacity: small signaling peptides are on aboard

**DOI:** 10.3389/fpls.2025.1679487

**Published:** 2025-10-13

**Authors:** Meixia Wang, Rong Lu, Zilin Zhang, Longxue Li, Yishui Chen, Huibin Han

**Affiliations:** ^1^ Jiangxi Provincial Key Laboratory of Conservation Biology, Jiangxi Agricultural University, Nanchang, China; ^2^ Research Center of Plant Functional Genes and Tissue Culture Technology, College of Bioscience and Bioengineering, Jiangxi Agricultural University, Nanchang, China; ^3^ Jiangxi Province Key Laboratory of Vegetable Cultivation and Utilization, Jiangxi Agricultural University, Nanchang, China

**Keywords:** CLE peptide, REF1 peptide, RLAF33, PSK, PORK1, BAM, plant regeneration

## 1 Introduction

**Figure 1:**
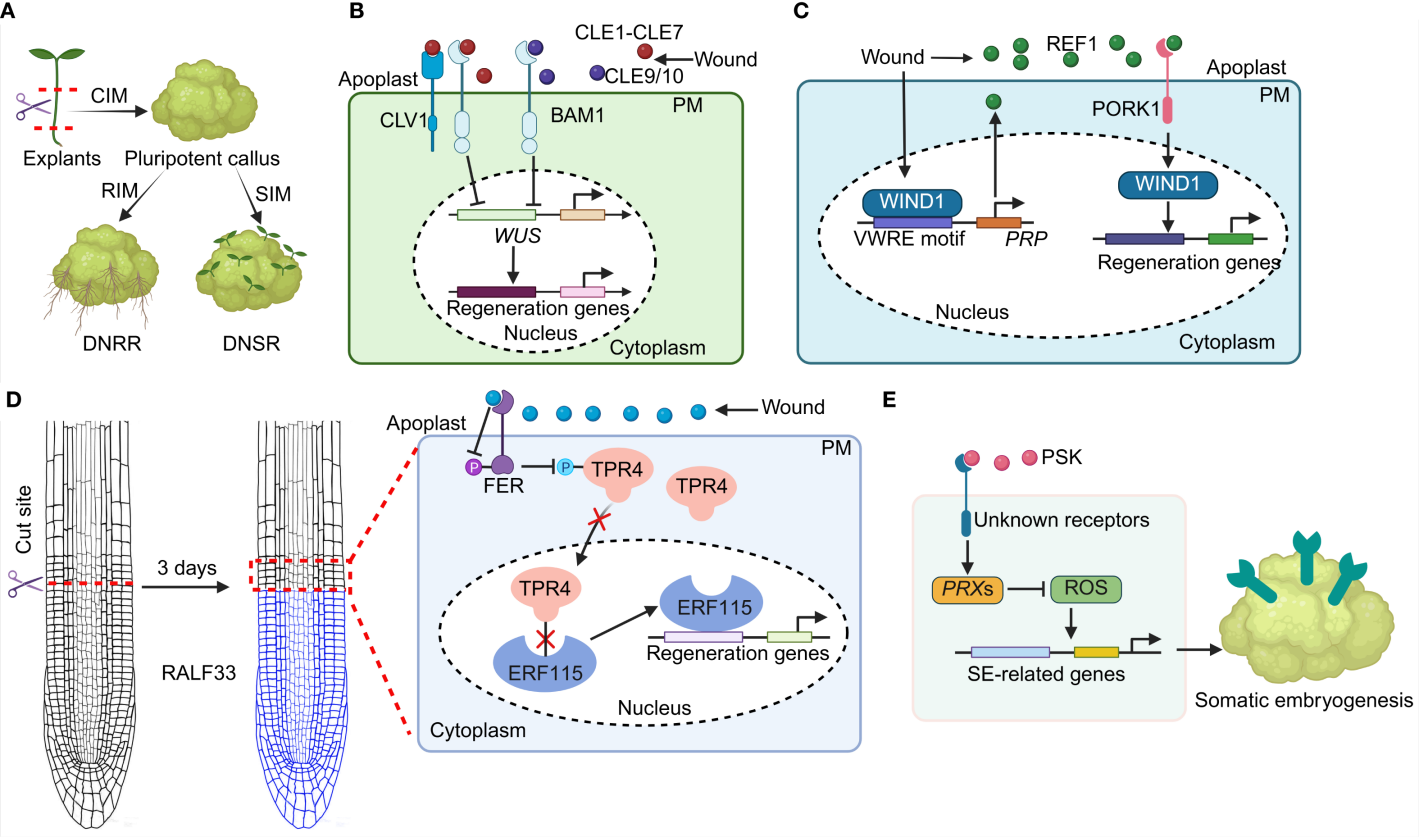
Small signaling peptides regulate plant regeneration potential. **(A)** A simplified plant DNSR and DNRR process. **(B)** CLE1-CLE7 and CLE9/10 peptides are perceived by the CLV1 and BAM1 receptors to regulate *WUS* transcription and its downstream targets of regeneration associated genes, thereby impeding shoot regeneration. **(C)** *WIND1* binds to the *PRP* promoter via the VWRE motif to initiate *PRP*-dependent REF1 peptide synthesis. The REF1-PORK1 module in turn induces the transcription of *WIND1*, thereby facilitating plant regeneration. **(D)** The RALF33-FER modulates the phosphorylation status of TPR4. The altered TPR4 protein then releases the ERF115 activity and promotes root regeneration. P: phosphorylation. **(E)** PSK peptide modulates SE development via regulating ROS signaling.

Plants exhibit a remarkable capacity to regenerate tissue, organs, or entirely new individuals after wounding or under *in vitro* conditions (Eshed Williams, 2021; Doll and Ikeuchi, 2025). Plant regeneration *in vitro* involves a biphasic process encompassing the acquisition of cell pluripotency and subsequent *de novo* shoots regeneration (DNSR) or *de novo* root regeneration (DNRR) (**Figure 1A**; Kim and Seo, 2025; Youngstrom et al., 2025). Initially, explants are cultured on an auxin-enriched callus-inducing medium (CIM) to induce the formation of a pluripotent callus. The pluripotent callus then undergoes extensive cellular reprogramming and spatial cell identities reorganization to generate shoots or roots (**Figure 1A**). Accumulating evidence indicates the vital involvement of phytohormones such as auxin, jasmonic acid (JA), cytokinin (CK), ethylene in regulating plant regeneration (García-Gómez and Ten Tusscher, 2024; Asghar et al., 2025; Xu and Yang, 2025). In addition, a couple of key transcription factors (TFs) such as WUSCHEL-RELATED HOMEOBOX (WOX), PLETHORAs (PLTs) have been reported to play a key role in plant regeneration (Islam et al., 2023; Chen et al., 2024). Although this regenerative capability has been well-documented across numerous plant species, the underlying mechanisms remain largely elusive (Eshed Williams, 2021; Chen et al., 2024).

Small signaling peptides represent a novel class of plant growth regulators, typically comprising fewer than 150 amino acids. These peptides are recognized by plasma membrane-localized receptors or co-receptors, which subsequently activate or deactivate specific regulatory pathways to modulate plant growth and stress adaptions (Ji et al., 2025; Xiao et al., 2025). Notably, several small signaling peptide-encoding genes, including *CLAVATA3*/*EMBRYO SURROUNDING REGION-RELATED* (*CLE*), *C-TERMINALLY ENCODED PEP TIDE* (*CEP*), *phytosulfokine* (*PSK*), and *GOLVEN*/*ROOT MERISTEM GROWTH FACTOR*/*CLE-LIKE* (*GLV*/*RGF*/*CLEL*) are differentially activated at specific stages of DNSR, as revealed by single-cell transcriptomic analysis, suggesting their potential roles in the plant shoot regeneration process (Zhai and Xu, 2021; Wang et al., 2022). Experimental data further demonstrate the regulatory functions of CLE1-CLE7 (Kang et al., 2022), CLE9/10 (Glazunova et al., 2025) and CLE46 (Ito et al., 2025), REGENERATION FACTOR1 (REF1) (Yang et al., 2024) and RAPID ALKALINIZATION FACTOR 33 (RALF33) peptides (Shen et al., 2025) in modulating plant regeneration capacity under *in vitro* conditions or in response to wounding signal as well as PSK peptides in the regulation of somatic embryogenesis (SE) (Hao et al., 2023; Luo et al., 2024) (**Table 1**; **Figures 1B–E**).

**Table 1:** A summary of reported role of small signaling peptides in plant regeneration.

Small peptide family	Receptors	Downstream targets	Function	References
CLE1-7	CLV1 and BAM1	WUS	Shoot regeneration	Kang et al., 2022
CLE9/10	BAM1	WUS	Shoot regeneration	Glazunova et al., 2025
CLE46	unknown	unknown	Root regeneration	Ito et al., 2025
REF1	PORK1	WIND1	Callus formation, shoot regeneration	Yang et al., 2024
RALF33	FER	TPR4, ERF115	Root regeneration	Shen et al., 2025
PSK	unknown	PRXs, ROS	Somatic embryogenesis	Hao et al., 2023; Luo et al., 2024

## 2 CLE-CLV1/BAM1 signaling module negatively regulates adventitious shoot regeneration

The *CLE* genes encode precursor proteins featuring an N-terminal signal sequence that targets them to the secretory pathway, a central variable region, and a highly conserved CLE domain at the C-terminus, which frequently undergoes posttranslational modifications to produce a functional polypeptide (Xie et al., 2022; Ji et al., 2025). Consistent with their pivotal functions in stem cell regulation (Selby and Jones, 2023; Shpak and Uzair, 2025), the expressions of *CLE* members are differentially activated at distinct phases of shoot regeneration (Zhai and Xu, 2021; Wang et al., 2022). Specifically, CLE1-CLE7 and CLE9/10 peptides have been demonstrated to negatively influence shoot regeneration (**Figure 1B**; Kang et al., 2022; Glazunova et al., 2025).

*CLE1*-*CLE7* gene expression is significantly induced by CIM or shoot-inducing medium (SIM), and CRISPR-engineered *cle1–7* septuple mutant exhibits an increased number of adventitious shoots (Kang et al., 2022). While most single *cle* mutants do not affect DNSR, the *cle4* and *cle7* mutants show enhanced DNSR (Kang et al., 2022). Conversely, application of synthetic CLE1–7 peptides exhibits a dose-dependent inhibition of DNSR, and overexpression of *CLE4* or *CLE7* also suppresses DNSR. In addition, CLE9/10 peptide also suppresses DNSR without affecting callus formation (**Figure 1B**; Glazunova et al., 2025).

Notably, the *clv1* and *bam1* mutants show increased shoot regeneration capabilities and are insensitive to CLE1-CLE7 peptide-mediated inhibition of shoot regeneration, confirming that CLAVATA1 (CLV1) and BARELY ANY MERISTEM1 (BAM1) receptors mediate the CLE1-CLE7 signal to negatively regulate DNSR. However, *clv1* mutant respond normally to CLE9/10 peptide treatment, while *bam1* mutant is insensitive to CLE9/10 peptide (Glazunova et al., 2025). Further studies reveal that the CLE-CLV1/BAM1 module restricts the transcriptional level of *WUSCHEL* (*WUS*), a critical regulator of plant regeneration (Ince and Sugimoto, 2023; Shpak and Uzair, 2025), thereby controlling shoot regeneration potential (**Figure 1B**).

## 3 REF1-PORK1 pathway promotes regeneration through activating *WIND1* expression

Recent studies have identified REF1, a Pep peptide homolog in tomato, as a crucial regulator of plant regenerative capacity (**Figure 1C**; Yang et al., 2024). The absence of *PRP*, the precursor of REF1 peptide, results in impaired callus formation and shoot regeneration, while *PRP* overexpression markedly enhances regenerative capacity (Yang et al., 2024). The exogenous application of synthetic REF1 peptide also enhances callus formation and shoot regeneration in wild-type (WT) plants in a dose-responsive manner, and compensates for the regenerative deficits observed in *prp* mutants. These findings collectively underscore the key role of REF1 peptide in regulating plant regeneration (Yang et al., 2024). Additionally, mutation in *PEPR1/2 ORTHOLOG RECEPTOR-LIKE KINASE 1* (*PORK1*) result in similar defects in callus formation and shoot regeneration that are observed in *prp* mutants. While *PORK1* overexpression significantly boosts regenerative capacity. Additionally, *pork1* mutant exhibits insensitivity to REF1 peptide treatments. Further investigations reveal that REF1 binds to PORK1, establishing PORK1 as the receptor for REF1 peptide (Yang et al., 2024).

WOUND-INDUCED DEDIFFERENTIATION 1 (WIND1), a prominent transcription factor implicated in plant regeneration (Iwase et al., 2017), operates downstream of the REF1-PORK1 module. Wounding signal significantly induces *WIND1* expression in WT tomato plants but not in *prp* and *pork1* mutants. The *wind1* tomato mutant exhibits severely compromised callus formation and shoot regeneration capacities, whereas *WIND1* overexpression plants show enhanced regenerative capabilities. Moreover, the regenerative deficiency in *wind1* mutants cannot be ameliorated by REF1 peptides. Importantly, wound-induced *WIND1* binds to the *PRP* promoter via the wound-responsive cis-element (VWRE) motif, promoting *PRP* transcription and amplifying REF1 signaling during regeneration. Notably, the utilization of the REF1 peptide has been shown to enhance both the regeneration and transformation efficiencies in soybean, wheat, and maize, indicating that REF1 peptide may substantially improve the recalcitrant crop regeneration and transformation processes. In conclusion, the REF1-PORK1-WIND1 module constitutes a regulatory loop that finely tunes the plant regeneration potential (**Figure 1C**; Yang et al., 2024).

## 4 RALF33-FER module regulates TPR4-ERF115 dynamics in root regeneration

RALF peptides, belonging to the cysteine-rich peptide family, are typically recognized by the *Catharanthus roseus* Receptor-Like Kinase 1-Like proteins (CrRLK1Ls), particularly FERONIA (FER) (Cheung, 2024; Pratyusha and Sarada, 2025). RALF peptides play a pivotal role in integrating developmental and environmental cues, thereby orchestrating optimal cellular and physiological responses (Cheung, 2024; Pratyusha and Sarada, 2025). Recent research highlights the significant function of the RALF33 peptide in root regeneration (**Figure 1D**; Shen et al., 2025).

RALF33 peptide shows rapid and substantial accumulation near the cut sites in the root within an hour post-resection, indicating its role as an early signaling molecule in response to root tip injury (Shen et al., 2025). Compared to WT plants, *ralf33* mutant exhibits reduced regeneration rates when cuts are made at the transition zone. Moreover, exogenous application of synthetic RALF33 peptide enhances regeneration rates. These findings collectively demonstrate that RALF33 acts as a positive regulator of root regeneration capacity. The *fer* mutant shows an increased regeneration capacity compared to WT plants. Furthermore, synthetic RALF33 peptide does not further enhance the regeneration rate in the *fer* mutant. Collectively, wounding induces the accumulation of RALF33 peptide, which subsequently suppresses FER activity, thereby enhancing root regeneration capacity.

Subsequent investigations have elucidated that TOPLESS-RELATED4 (TPR4) and the transcription factor (TF) ERF115 act downstream of the RALF33-FER signaling module in root regeneration processes. FER interacts with TPR4, and this interaction inhibits the localization of TPR4 in nucleus. The mis-localized TPRE4 is unable to suppress the activity of ETHYLENE RESPONSE FACTOR 115 (ERF115), a critical regenerative TF (Zhou et al., 2019; Matosevich et al., 2020). The release of ERF115 then activates its downstream targets, thereby impairing root regeneration. (**Figure 1D**; Shen et al., 2025).

In addition, *CLE46* is reported to have an inhibitory effect on DNRR originating from leaf explants (Ito et al., 2025). Spatial expression analysis indicates that the activity of the *CLE46* promoter-GUS fusion is confined to the shoot apex in young Arabidopsis seedlings. Upon the excision of petioles in leaves, *CLE46* activity is induced in response to wound, exerting a negative regulatory effect on root regeneration. This is evidenced by the increased root regeneration rate observed at the leaf cut end in the *cle46* mutant. In hypocotyl-excised seedlings, *CLE46* activity remains uninduced, while *cle46* mutant still displays a significantly higher root regeneration rate compared to WT seedlings. Collectively, these findings suggest that *CLE46*, expressed in the shoot apex, suppresses root regeneration in the lower regions of the plant tissue (Ito et al., 2025). However, the molecule mechanism of CLE46-mediated DNRR regulation requires further investigations.

## 5 PSK peptide regulates somatic embryogenesis

Somatic embryogenesis (SE) is widely employed for the transformation and regeneration of diverse plant species (Martínez and Corredoira, 2024), this process is regulated phytosulfokine (PSK) peptide (Hao et al., 2023; Luo et al., 2024). Application of synthetic PSK peptide markedly stimulates the transitions of proembryogenic masses (PEMs) and enhances SE development in *Cunninghamia lanceolata* in a genotype-independent manner (Hao et al., 2023). Transcriptomic analyses reveal that PSK treatment results in a disruption of reactive oxygen species (ROS), specifically by reducing H_2_O_2_ levels through modulation of *PEROXIDASE*s (*PRX*s) expression. This reduction in ROS promotes the expression of SE-related genes, including *WUS*, *WUSCHEL HOMEOBOX 2* (*WOX2*), *BABY BOOM* (*BBM*), *AINTEGU MENTA* (*ANT*) in the early phases of SE induction (Hao et al., 2023). Consistent with these findings, the overexpression of *ClPSK*, a homolog of *PSK* genes in *Cunninghamia lanceolata* (Wu et al., 2019), also facilitates enhanced SE induction and decreases ROS levels (Hao et al., 2023). PSK peptide can also trigger SE formation in *Pinus massoniana* by lowering H_2_O_2_ concentrations (Luo et al., 2024). Collectively, these results underscore the pivotal and conserved role of PSK peptide in the promotion of SE may via a genotype-independent manner (**Figure 1E**).

## 6 Future perspectives

Genetic transformation in plants is essential for advancing crop enhancement and commercial cultivation. Nevertheless, the issue of limited plant regeneration capacity continues to be a critical impediment in the precise generation of genetically modified plants (Mei et al., 2024; Wang et al., 2025; Youngstrom et al., 2025). Although numerous genes, such as *BBM*, *GROWTH-REGULATING FACTOR*s (*GRF*s), and *WUS*, have been identified in promoting the regeneration of transgenic or gene-edited plants (Youngstrom et al., 2025). Nonetheless, these conventional approaches are frequently genotype-dependent and constrained by plants’ inherent recalcitrance to low regeneration capacity. The discovery of small signaling peptides in plant regeneration holds a potential to overcome this limitation. However, several questions need to be addressed to elucidate the underlying molecular mechanisms, thereby facilitating their widespread application in agriculture.

(1) How does wound signaling orchestrate the biosynthesis and post-translational modifications to yield functional peptides? Tissue injury triggers the synthesis of CLE1-7, CLE9/10, CLE46, REF1, RALF33 and PSK peptides, thus initiating regenerative processes (**Figure 1**), though the precise activation mechanisms remain elusive. It has been documented that proteolytic processing, proline hydroxylation, and arabinosylation are essential for the formation of an active CLE peptide (Stührwohldt et al., 2020a, 2020b). Key enzymes involved in CLE peptide processing and post-modification include SUBTILASEs (SBTs), PROLYL-4 HYDROXYLASE (P4H), O-arabinosyltransferase (HPAT), and arabinosyl transferases such as REDUCED RESIDUAL ARABINOSE 3 (RRA3) and XYLOGLUCANASE113 (XEG113) (Olsson et al., 2019; Stührwohldt and Schaller, 2019). RALF peptides, characterized by multiple cysteine residues, necessitate proper disulfide bond formation for their activity and receptor binding (Frederick et al., 2019; Moussu et al., 2020). The plant disulfide isomerase OaPDI, isolated from *Oldenlandia affinis*, facilitates the formation of disulfide bonds, thereby generating bioactive peptides (Gruber et al., 2007). Furthermore, metacaspases (MCs) and SUMOylation have been implicated in the proteolytic processing of PROPEPs, the precursors of Pep peptides (Hander et al., 2019; Shen et al., 2019; Zhang et al., 2025), leading to the production of functional Pep peptides. It is imperative to elucidate how wounding activates these enzymatic pathways to generate functional small signaling peptides that finely regulate the regenerative processes.

(2) What is the interplay between small signaling peptides and phytohormonal signaling pathways in plant regeneration? It is well-established that phytohormones are crucial in the plant regeneration process (García-Gómez and Ten Tusscher, 2024; Asghar et al., 2025; Xu and Yang, 2025). Additionally, small signaling peptides are responsive to phytohormones (Ji et al., 2025; Xiao et al., 2025). Specifically, jasmonate (JA) and auxin modulate root regeneration via ERF115 (Zhou et al., 2019), indicating that RALF33 may potentially integrate JA and auxin signaling to regulate root regeneration. These data further suggest a possible crosstalk between small signaling peptides and phytohormones in plant regeneration. However, the exact mechanisms remain to be clarified. Furthermore, what are the dynamics of these small signaling peptides, as they could induce either antagonistic or synergistic effects in plant regeneration processes? A comprehensive analysis of these intricate interactions in future research will aid in their effective application in agricultural practices.

(3) How do small signaling peptides modulate epigenetic mechanisms during plant regeneration? At the cellular level, the regeneration process entails dynamic alterations in gene expression that redirect cell fate transitions. Notable epigenetic modifications implicated in plant regeneration include DNA methylation, trimethylation at lysine 27 of histone H3 (H3K27me3), trimethylation at lysine 4 of histone H3 (H3K4me3), and acetylation of histones H3 and H4 (H3/H4 acetylation) (Chen et al., 2023; Li et al., 2024). During this process, the expression of *WUS* is subject to complex epigenetic regulation involving both DNA methylation and histone modifications. The *WUS* promoter is methylated in both CG and CHG contexts (Li et al., 2011; Shemer et al., 2015), and its activation is facilitated by the removal of the repressive histone mark H3K27me3 (Zhang et al., 2017). The H3K9 methyltransferase KRYPTONITE (KYP), the H3K4 demethylase JUMONJI 14 (JMJ14), and the histone acetyltransferase HISTONE ACETYLTRANSFERASE OF THE CBP FAMILY 1 (HAC1) have all been shown to regulate *WUS* expression (Li et al., 2011). Additionally, the rapid induction of *WIND1* following wounding is orchestrated by elevated levels of H3K9/14 acetylation and H3K4 trimethylation (Rymen et al., 2019). Histone acetyltransferases such as ACETYLTRANSFERASES1 (HAG1), HAG3, and General Control Non-depressible 5 (GCN5) also play roles in the transcriptional regulation of *WIND1* (Rymen et al., 2019). POLYCOMB REPRESSIVE COMPLEX 2 (PRC2) facilitates the repression of H3K27me3 to enhance *WIND1* transcription (Ikeuchi et al., 2015). Nevertheless, the precise mechanisms by which small signaling peptides influence these epigenetic regulations to reprogram the transcriptional landscape of key regulators in plant regeneration warrant further investigation. The deployment of advanced epigenetic methodologies such as CRISPR-based activation/interference/epigenetic systems (Goell and Hilton, 2021; Cai et al., 2023; Joshi and Wang, 2024), will facilitate the construction of unprecedented transcriptional networks that are mediated by small signaling peptides in plant regeneration processes.

(4) How to boost plant regeneration capacity by engineered small signaling peptides? Genetic modifications of peptide precursors have proven to ensure optimal plant growth and development under fluctuating environmental conditions. Nonetheless, the extraction of endogenous small signaling peptides remains technically challenging due to their exceedingly low concentrations in planta. The exogenous application of chemically synthesized small signaling peptides offers a straightforward and efficient approach to modulate plant plasticity. Previous studies have shown that PSK peptide analogues incorporating diastereomers or *N*-methylation can enhance regeneration efficiency (van de Sande et al., 2024). Moreover, antagonistic CLE peptides have been developed by introducing an amino acid substitution at the sixth conserved glycine residue within the CLE motif (Song et al., 2013; Czyzewicz et al., 2015). Additionally, such amino acid substitutions in the CLE motif result in the generation of non-natural CLE-like peptides with bifunctional properties (Hirakawa et al., 2017). Consequently, there is considerable interest in creating more effective peptide variants, such as agonists, antagonists, chemically modified peptides, or non-natural peptide-like molecules, to enhance plant regeneration potential through targeted chemical modifications or substitutions. High-performance liquid chromatography (HPLC) or synthetic biology (Wu et al., 2021; Ji et al., 2025) could be utilized to produce these bioactive modified small signaling peptides. Additionally, the efficient delivery of small signaling peptides into plant systems presents a significant challenge. To address this issue, the development of nanomaterials (Santana et al., 2020), hydrogels (Zhang et al., 2023), and viral vectors (Abrahamian et al., 2020) may provide effective strategy for encapsulating and transporting small signaling peptides into plant tissues. The integration of these innovative techniques would enhance the application of small signaling peptides in the precise molecular breeding of crops and horticultural plants by improving regeneration and transformation efficiency.
